# Zeolites: A series of promising biomaterials in bone tissue engineering

**DOI:** 10.3389/fbioe.2022.1066552

**Published:** 2022-11-17

**Authors:** Yue Li, Yanting Cai, Tianyan Chen, Xingfu Bao

**Affiliations:** Department of Orthodontics, Jilin Provincial Key Laboratory of Tooth Development and Bone Remodeling, School and Hospital of Stomatology, Jilin University, Changchun, China

**Keywords:** zeolites, bone tissue engineering, biomaterials, biomedical applications, scaffold

## Abstract

As an important worldwide medical issue, bone defect exhibits a variety of physical and psychological consequences on sufferers. Some features of clinical treatments including bone grafting and limb shortening are not satisfactory. Recently, bone tissue engineering has been considered as the most effective approach to dealing with the issue of bone deformities. Meanwhile, a variety of biomaterials have been rationally designed and created for the bone regeneration and tissue repairing. Among all these admirable biomaterials for bone remodeling, zeolite-based materials can serve as efficient scaffold candidates with excellent osteo-inductivity. In addition, the porous nature and high biocompatibility of zeolites endow them with the ability as ideal substrates for cell adhesion and proliferation. More importantly, zeolites are investigated as potential coating materials for implants because they have been proven to increase osteo-conductivity and aid in local elastic modeling. Last but not least, zeolites can also be used to treat bone disorders and act as dietary supplements during the practical applications. Accordingly, numerous benefits of zeolite prompt us to summarize their recent biomedical progress including but not limited to the distinguishing characteristics, broad classifications, as well as promising usages in bone tissue engineering.

## 1 Introduction

**FIGURE 1:**
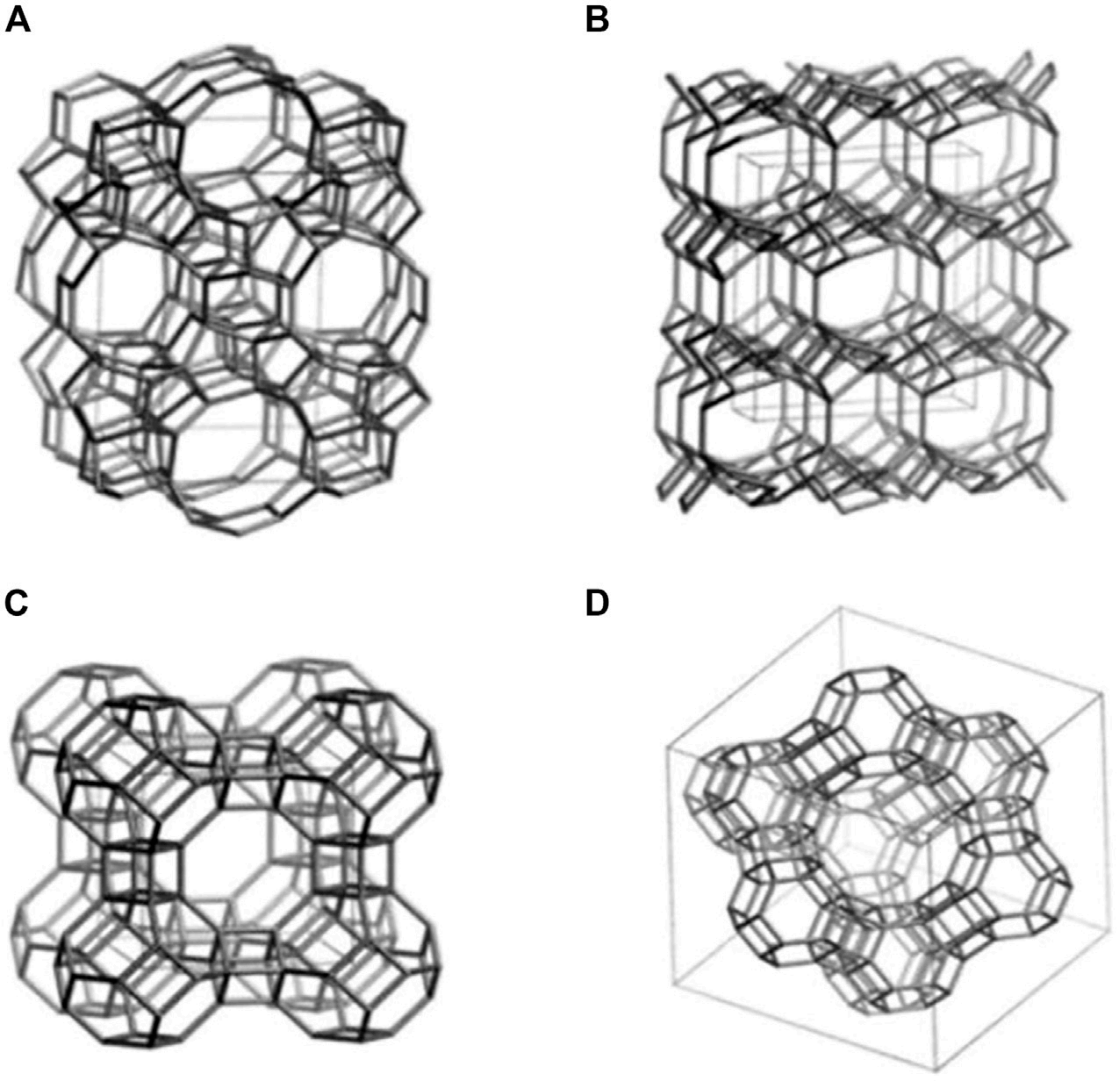
Structural models of several types of zeolites including **(A)** ZSM-5, **(B)** Clinoptilolite, **(C)** Zeolite A, and **(D)** Zeolite Y (Servatan et al., 2020).

Zeolites are a type of crystalline aluminosilicate mineral comprised of hydrated tectosilicates containing silicon and aluminum atoms (Derakhshankhah et al., 2020). Tetrahedral unit (TO_4_) connected to an oxygen atom in a three-dimensional framework is the basic building component of zeolites. The presence of Si and Al, both of which are tetravalent in this context, accounts for the lattice’s negative charge. Exchangeable cations including sodium, potassium, or calcium must be present to neutralize the negative charge. The remaining spaces in the porous structure are filled with aqueous moisture (Bacakova et al., 2018). TO_4_ tetrahedra (for example, [SiO_4_]^4-^, [AlO_4_]^5-^) are linked together by oxygen atoms to produce zeolitic frameworks (Zarrintaj et al., 2020). As a result of the spatial connection between SiO_4_ and AlO_4_ tetrahedra in a three-dimensional system, the structure has well-defined holes and chambers (Yao et al., 2018). The chemical structure of several zeolites is depicted in Figure 1 (Servatan et al., 2020). Owing to the unique structure, zeolite has been applied in a variety of biological and medical applications.

**FIGURE 2:**
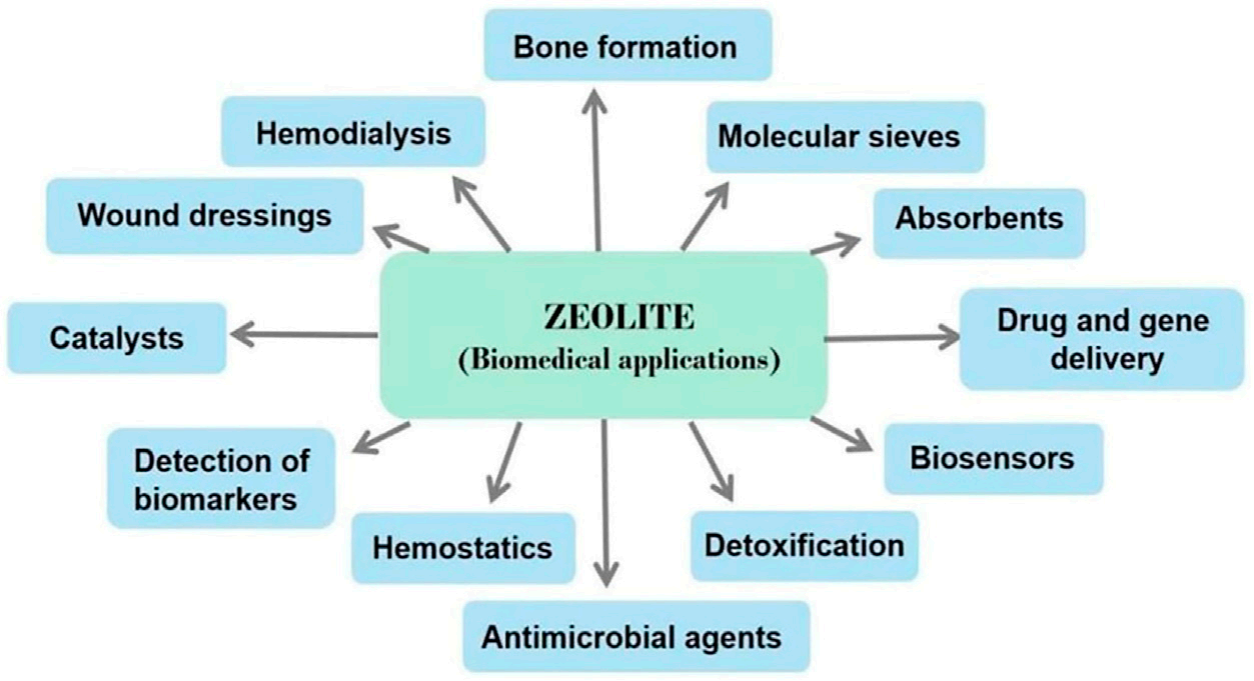
A summary of zeolites used in biomedical fields.

Even though the International Zeolite Association (IZA) database lists more than 200 different zeolitic structures, only a handful of zeolites are anticipated to have widely applicable and accessible structures. Different three-letter designations were assigned to zeolites based on their diverse structures, which were distinct from their common or commercial names, such as Mordenite Framework Inverted (MFI), Ferrierite (FER), and Heulandite-type zeolite (HEU) (Zarrintaj et al., 2020). For the purpose of highlighting the systems and properties of zeolites, a variety of classification approaches have been developed. Zeolites can be classified into low silicon, medium silicon, high silicon, and pure silicon zeolite based on the silicon-aluminum ratio, where Si/Al <1 is low silicon, 1.5 < Si/Al<10 is medium silicon, Si/Al > 10 is high silicon, and Si/Al = ∞ is pure silica zeolite. Furthermore, because zeolites have a homogeneous and regular porous structure, they can be separated into two types based on the distribution of pores: one-dimensional channel structure and three-dimensional cross channel structure. Finally, since zeolites hold a specific pore size range, they can be categorized into microporous, mesoporous, and large pore channels. Pore sizes less than 2 nm are considered as micropores, pore sizes between 2 nm and 50 nm are considered as mesopores, and pore sizes greater than 50 nm are considered as giant pore. However, the most prevalent method is to classify zeolites according to their origin. Thus, synthetic zeolites are distinguished from natural zeolites (Eroglu et al., 2017). In the past 50 years, natural zeolites have developed rapidly, particularly in commercial manufacturing. Volcanic deposits in an alkaline environment frequently yield naturally occurring zeolites with a range of structural configurations. Clinoptilolite and erionite are examples of naturally occurring zeolites that have been utilized in industrial settings. Clinoptilolite is a natural zeolite that is most frequently used (Amiri-Yazani et al., 2019). Due to their diverse chemical compositions, high cost, and irregular structure, natural zeolites are difficult to use on a large scale. As a result of developments in laboratory synthesis techniques, synthetic zeolites are now suitable for widespread commercial use. The MFI structure is one of the most significant zeolite sales in terms of commercialization. The Mobil Oil Company developed Mobil Zeolite Socony Mobil-5 (ZSM-5) as an MFI in 1975. ZSM-5 is one of the most catalytic zeolites, and it can catalyze chemical reactions within the body (Dai et al., 2022). Based on these porous structure, absorption capacity, and other advantageous properties, zeolite can be utilized in various biological applications depicted in Figure 2.

**FIGURE 3:**
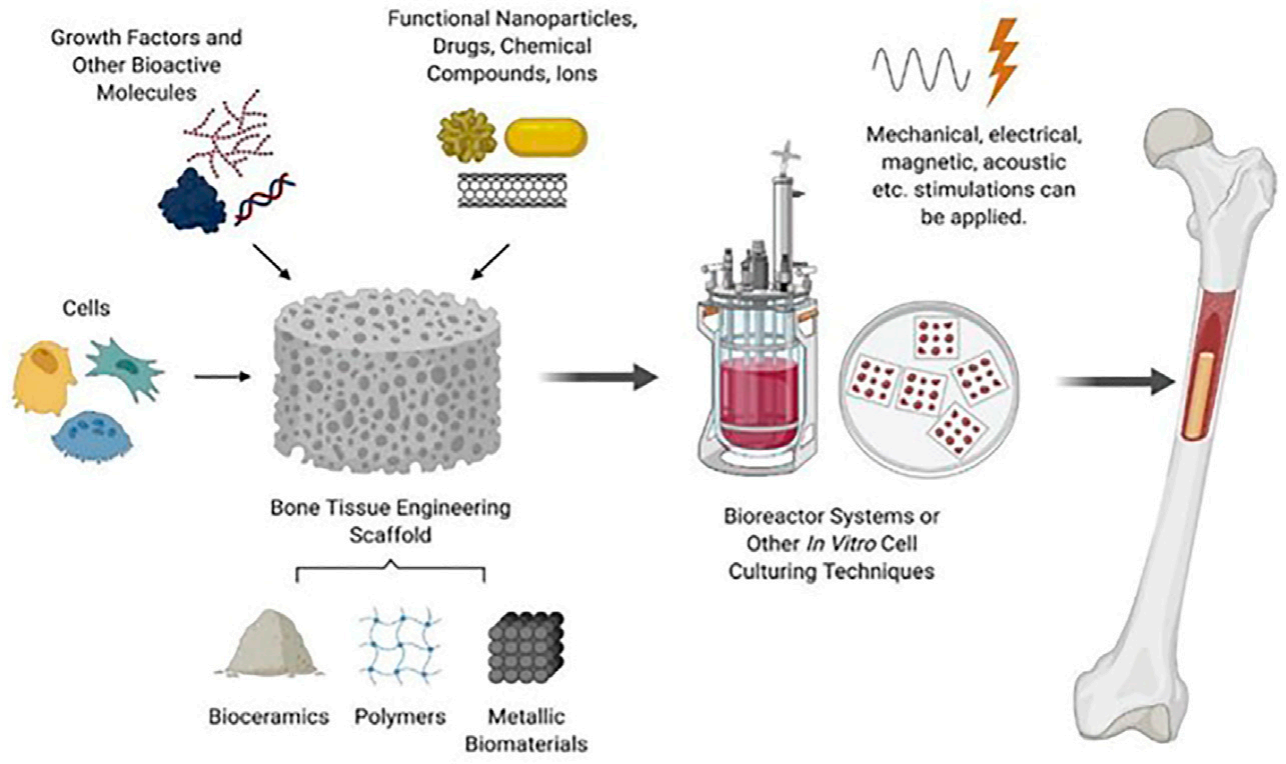
Bone tissue engineering strategy including scaffold, cells and molecules (Collins et al., 2021).

Bone defect has been the major clinical issues and has a substantial effect on society. The most common causes of bone defect are infections, trauma, and degenerative bone diseases including osteoarthritis (OA), rheumatoid arthritis (RA), and bone tumors. Promoting bone structure recovery and bone regeneration is the most essential clinical treatment strategy. To treat bone defects, autografts, allografts, and xenografts are utilized. The gold standard for bone regeneration is autografts, but they have limitations. Firstly, insufficient donor site resources or problems may exist. Secondly, autografts are incapable of correcting major deformities and can cause pain, infection, and wound healing. Thirdly, allografts and xenografts pose a greater threat to graft survival than autografts and immune rejection. Thus, these limitations demand innovative bone-repair techniques (Zarrintaj et al., 2020). As a result, bone tissue engineering developed rapidly recent years. A superior tissue engineering structure must contain a scaffold, cytokines, and seed cells. Scaffolding materials are essential to the construction of artificial bones as they act as the physical foundation. Ideal scaffolds must have properties similar to those of natural bone, promoting adhesion, proliferation, migration, differentiation, and angiogenesis of seed cells. They must be incorporated into bone tissue during the healing process and support a normal load (Diomede et al., 2020; Lin et al., 2020). To overcome the limitations of current bone tissue engineering techniques, researchers are examining biomimetic scaffolds. Figure 3 illustrates the distinctions between bone tissue engineering scaffolds and autografts, allografts, and xenografts (Collins et al., 2021). In addition to the scaffold, cell behavior is an important factor in bone regeneration. Recruitment of osteoblast precursors follows osteoclast-mediated bone resorption. These cells then form osteoblasts to replace bone that has been absorbed. In this perspective, the knowledge regarding bone metabolism acquired from bone abnormality disease can be applied in bone tissue engineering (Zheng et al., 2019; Amirazad et al., 2022). Additionally, implants are broadly employed in clinical applications to facilitate the bone tissue engineering. In clinical practice, metal materials such as titanium alloy is commonly used to fix the scaffold and provide enough space for bone growth. However, the corrosion of metal appears commonly and lead to the loose of scaffold. Even though Ti6Al4V is biocompatible after implantation, it releases Al and V ions into the surrounding environment as a result of corrosion. Vanadium ions are cytotoxic, whereas aluminum ions can lead to neurological disorders, posing a significant health risk to the patient. Finally, there is a modulus mismatch between Ti6Al4V and bone, as the elastic modulus of Ti6Al4V (110 GPa) is significantly greater than that of bone (10–40 GPa). This difference can put pressure on bone tissue, which can lead to loosening of the implant and failure of osteointegration (Bedi et al., 2009a; 2009b). Considering the challenges mentioned above, zeolites’ unique structure makes them ideal for bone regeneration. In this review, we summarized the recent literature and reviewed the progress of zeolites in bone-related applications.

## 2 Applications of zeolites in bone tissue engineering

Along with their porosity, zeolites have been the subject of extensive research in the field of bone tissue engineering. On the one hand, zeolite scaffolds can be structured to have the highest mechanical strength to withstand compression from adjacent bone for large bone defects, thereby improving osteo-conductivity. Zeolites, on the other hand, have been shown to improve cell adhesion and proliferation, thereby increasing osteo-inductivity. Besides that, zeolite coatings are highly biocompatible and can reduce local inflammation due to their ability to prevent the escape of harmful ions into the surrounding tissue. Thus, it can modify the surface of synthetic materials. Generally, because of their stable biological properties, different topologies, and high osteogenic potential, zeolites are good candidates for bone tissue engineering. There is evidence that nanoscale topology can significantly influence the biological performance of scaffolds due to the high surface area, high protein adsorption, and different topology lead to the different purpose of cell behavior (Zarrintaj et al., 2020).

### 2.1 MFI

The unique characteristic like consistent pore size and excellent corrosion resistance grant MFI various function which are needed in the bone tissue engineering. Methodical optimization process can be used to change the porosity, topology, morphology, wettability, roughness, and charge of MFI parameters. The most thoroughly studied and developed option is the MFI zeolite membrane, which is produced in a single layer to maximize the material’s corrosion resistance and function as a barrier to prevent harmful ions from escaping. The most prominent MFI zeolite materials have been shown to be chemically and thermally stable, allowing them to be used in drug administration and hydroxyapatite production (Doubkova et al., 2019). *In vivo* studies, MFI also exhibited high biological compatibility and extraordinary osteoconductive and osteo-inductive properties, promoting the formation and growth of new bone tissue (Bedi et al., 2009b).

#### 2.1.1 Corrosion-resistance

Certain biomaterial qualities, such as bioactivity and biocompatibility, resistance to corrosion and abrasion, must be considered during the designing of bone implants in order to make alloplastic bone implants economically viable and avoid implant failure. Because of their superior mechanical properties, metals and their alloys are the most commonly utilized biomaterials in bone-implant engineering. However, with wear and tear, some of them may produce potentially poisonous Co., Cr, Al, Ni, or V ions. As a result, dangerous discharges must be avoided in order to prevent implant failure (Purnomo et al., 2020). Rajwant S. Bedi et al. produced a new type of inorganic corrosion-resistant zeolite coating on aluminum alloys, and the coating was shown to be extremely corrosion resistant with good adhesion and wear resistance even in pitting aggressive medium or strong acid (Cheng et al., 2001). Then Bedi et al. created zeolite MFI coatings on the Ti6Al4V substrate using high silica zeolite ZSM-5. MFI coating can give excellent adherence to metal surfaces while also preventing harmful ions such as Al and V from entering tissues. Ti6Al4V substrate with MFI coating shows superior corrosion resistance than bare titanium alloy in 0.856 M NaCl solution at pH 7.0 and 1.0. (Bedi et al., 2009a). Aside from ZSM-5, silicalite-1 is another type of zeolite having an MFI structure and a regular pore structure. Because silicalite-1 is a fully siliceous homologue of the pentasil zeolite and contains no Al, it may escape the harmful influence of Al^3+^ on humans and is safer for their bodies. In fact, silicalite-1 film as a type of zeolite coating on titanium alloy has been demonstrated to be a non-corrosive siliceous porous coating with the function of facilitating cell adhesion and calcium deposition.

#### 2.1.2 Drug delivery

As a kind of zeolite, MFI has the function of drug loading because they are an inorganic porous crystalline substance with a highly organized structure. The mesopores and micropores among the MFI nanocrystals not only supplied large specific surface areas for adsorbing more drug molecules, but can also operate as a carrier to gradually release them along with body fluid circulation, maximizing drug activity. Although most implant materials are nontoxic and nonimmunogenic, implants frequently produce “foreign body reactions,” which may end in implantation failure due to immunological rejection. So a medicine is required to prevent implant loosening; more critically, it must release gradually and be highly stable. Accordingly, researchers constructed various vectors with transition metal on the basis of ZSM-5 and MOR zeolite, which are proved can absorb famotidine (Tavolaro et al., 2013). In another research, researchers created pifithrin-α (PFTα)-loaded ZSM-5/chitosan (ZSM-5/CS/PFTα) ellipsoids. PFTα is an inhibitor to suppress the gene function of p53. The biological features of ZSM-5/CS/PFTα ellipsoids are investigated utilizing human bone marrow stromal cells (hBMSCs) as a cell model and ZSM-5/CS as a control group. Both the ZSM-5/CS and ZSM-5/CS/PFTα ellipsoids show excellent cytocompatibility, which enhances cells spreading, adhesion, and proliferation (Zhu et al., 2017a). In the same year, they replaced PFTα with SC79 and created SC79-loaded ZSM-5/chitosan (ZSM-5/CS/SC79) porous scaffolds for the first time by freeze-drying ZSM-5/CS porous scaffolds and then loading SC79 medicinal molecules. The ZSM-5/CS scaffolds were porous in three dimensions, and the ZSM-5 ellipsoids were evenly distributed on the CS films. The micropores and mesopores within and among the ZSM-5 crystals can function as routes for loading SC79, as they did in their previous study. *In vitro* cell experiments revealed that both the ZSM-5/CS and ZSM-5/CS/SC79 scaffolds could promote human bone mesenchymal stem cells adhesion, spreading, and proliferation. Furthermore, *in vivo* animal experiments showed that SC79 released from the ZSM-5/CS/SC79 scaffolds could boost new bone regeneration without any systemic negative effects (Zhu et al., 2017b).

#### 2.1.3 Promoting bone formation

Many investigations on the effects of zeolites on bone formation have been conducted. To summarize, the mechanism consists mostly of three aspects: The first is zeolite improves implant hydrophilicity during bone formation; a more hydrophilic surface can give greater cellular adhesion for osteoblasts during bone formation. The second is that zeolite topologies provide a large surface area for cell adhesion and hydroxyapatite (HAP) crystalline (Hunter et al., 1995; Zanello et al., 2006). The third is that zeolite facilitate HAP formation, which is physically and chemically identical to the mineral components of human bones, thus enhancing biocompatibility and osteo-conductivity. As a kind of zeolite, MFI also possess the above characteristics of zeolites. For MFI coatings, zeolite crystal size ranged between 1 and 5 mm (Bedi et al., 2009b). The ZSM-5 type zeolite have MFI type structure, with 350 nm in diameter and 165 nm in thickness. In the biocompatibility test, the hBMSCs had an ideal adhesion and proliferation on the surface of ZSM-5. (Guo et al., 2014).

**FIGURE 4:**
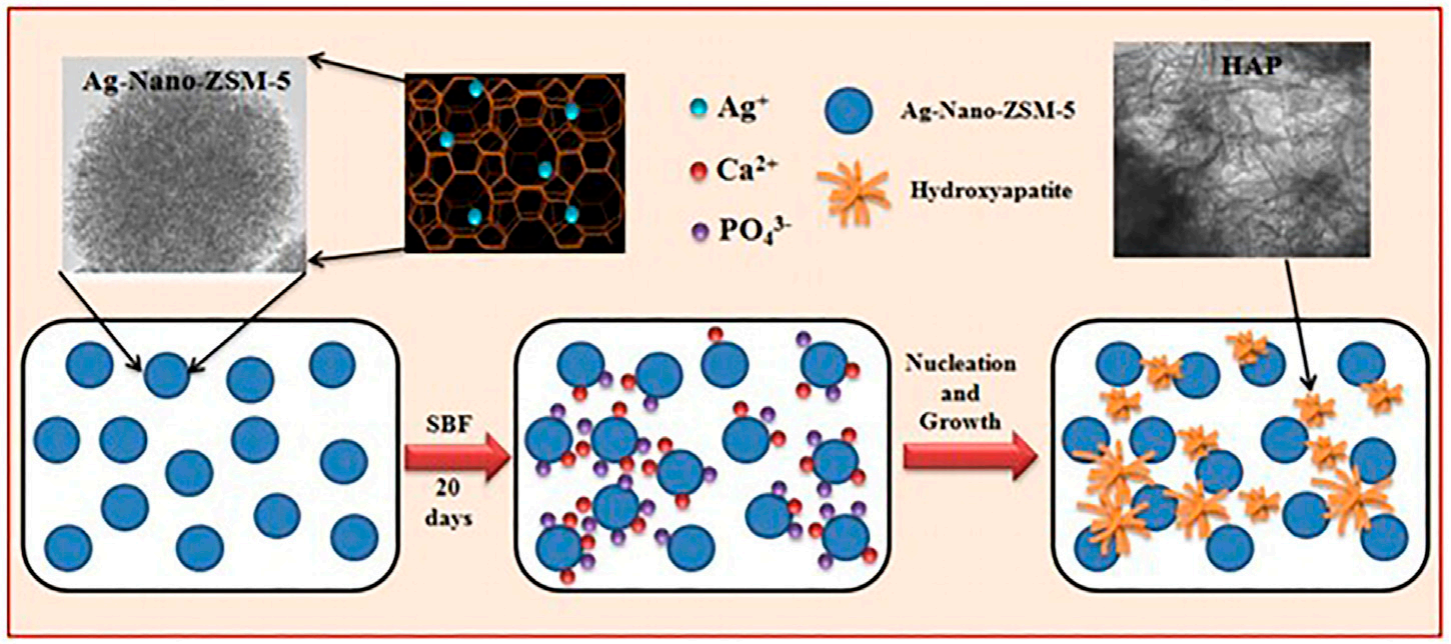
The process of HAP formation in Ag-Nano-ZSM-5 in simulated body fluid (Kaur et al., 2015).

A number of studies have been conducted in order to promote the creation of HAP so as to aid in the growth of bone tissue. Researchers created ZSM-5 type zeolite using a non-organic template, functionalized the surface with Ag^+^ or Zn^2+^, and investigated the materials’ bioactivity *in vitro* in Simulated Body Fluid (SBF). A homogenous and dense bonelike apatite layer formed on the functionalized zeolite surface in less than 14 days. The elements Ca, *p*, and Si were mostly detected by Energy Dispersive Spectrometer (EDS), and the peak of Ca and *p* visibly increased over time. These findings reveal that metal functionalized ZSM-5 type zeolites have an exceptional ability to induce apatite synthesis even in the absence of cells, which can considerably aid in bone development. Gallium ions can also be used to prevent bone desorption and cure osteoporosis by boosting the mineralization of phosphorus and calcium bone apatite. A bioactive ZSM-5 [Ga] type zeolite was created and functionalized with Ca^2+^ ions (Sánchez et al., 2013). After 7 days of immersion in SBF, a layer of apatite was formed on the ZSM-5 [Ga] type zeolite. Furthermore Balwinder Kaur et al. produced silver ion-exchanged conventional ZSM-5 (Ag-ZSM-5) and nanocrystalline Nano-ZSM-5 (Ag-Nano-ZSM-5) materials. These two types of zeolites were immersed in SBF for various time durations to develop HAP nanoparticles. In comparison to Ag-ZSM-5, a large amount of HAP was produced in the Ag-Nano-ZSM-5 matrix after incubation. Figure 4 depicts the mechanism for HAP growth in the Ag-Nano-ZSM-5 matrix (Kaur et al., 2015).

Foam ceramics are another type of commonly utilized bone healing material that can enhance the production of HAP. Hao et al. created silicalite-1/SiC foam and ZSM-5/SiC foam by doping ZSM-5 and silicalite-1 on the surface of foam ceramics. The bioactivity of ZSM-5/SiC foam and silicalite-1/SiC foam was comparable, and they were harmless to primary osteoblasts. Ca, *p*, and O elements were found in the deposit sediment on ZSM-5/SiC foam and silicalite-1/SiC foam, which corresponded to an apatite-like layer. Bone-like apatite is generated during the development and growth of bones. According to the zeta potential studies, ZSM-5/SiC foam and silicalite-1/SiC foam had higher negative charges on the surface than SiC foam. Negative surface charges tend to interact with cations in the solution, such as Ca^2+^, to create amorphous or nanocrystalline calcium phosphate with a positive charge on the surface. Then they attract anions (like PO_4_^3−^). Calcium and phosphorus nucleation occurs as local oversaturation increases, crystals progressively develop, and eventually a bone-like apatite layer form. Furthermore, as compared to SiC foam, ZSM-5/SiC foam and silicalite-1/SiC foam may result in enhanced proliferation, differentiation, and adhesion of osteoblasts following incubation. The 3D microporous structure and complex microcrystal topology provide sufficient surface area and attachment sites for Ca^2+^, as well as osteoblast adhesion, differentiation, and proliferation (Hao et al., 2019).

Another way zeolites enhance bone formation is regulating cell proliferation, adhesion and gene expression to increase bone metabolic activity. MFI coating has a similar elastic modulus to bone and can effectively prevent bone resorption. Furthermore, it can aid in the maintenance of cells growth and osteogenic differentiation *in vitro* (Bedi et al., 2009a). In another study, they discovered that zeolite MFI had osteo-conductivity and osteo-inductivity properties, as well as the ability to promote osteogenesis, mineralization, and the expression of osteoblast genes (Bedi et al., 2009b). After that, the same scientist coated the titanium implant with zeolite MFI-hydroxyapatite, enhancing osteo-induction and osteo-conduction, which can speed up the recovery and healing process. One evidence of osteo-inductivity augmentation is the considerable rise in BMP-2 gene with the ability of triggering osteogenic differentiation of bone cell precursors (Bedi et al., 2012).

**FIGURE 5:**
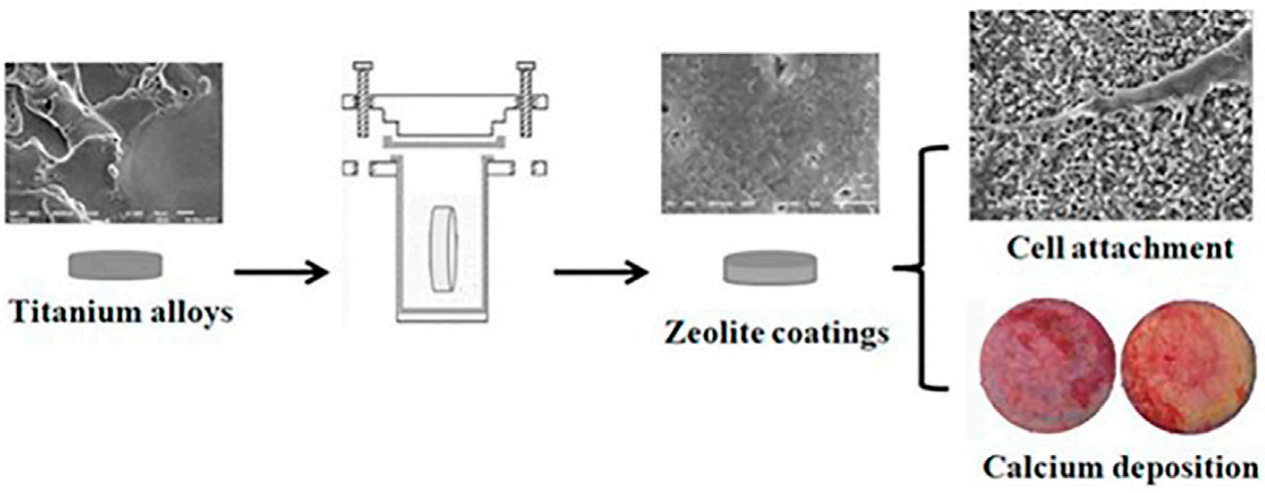
Zeolite coatings on titanium alloys which could improve the cell attachment and facilitate the bio-mineralization (Li et al., 2019).

Further researches were conducted based on previous results. A rabbit femur implant model was used in this investigation to see if the MFI zeolite covering may improve implant osteointegration and osteogenesis. The *in situ* crystallization process was used to create an MFI coating on a Ti6Al4V substrate in this investigation. Figure 5 shows the process of synthesizing calcium incorporated zeolite coatings and the ability of promoting cell attachment as well as calcium deposition for zeolite coatings. These findings indicate that MFI-coated Ti6Al4V (M-Ti) showed higher bioactivity *in vivo*. Cell differentiation was studied in addition to cell adhesion and proliferation. The results of ALP staining and activity assays at 4 and 7 days indicated that the MFI coating increased the expression of ALP, an osteogenic differentiation marker. The results showed that the M-Ti group had superior osteointegration than the Ti group at 4 and 12 weeks, and the BA values showed that the M-Ti group was actively forming new bone. The findings revealed that MFI coating had comparable biocompatibility to Ti6Al4V substrates while exhibiting better osteogenic differentiation, improved osteointegration, and osteogenesis of the scaffolds *in vivo* (Li et al., 2015). Doubkova et al. modified the material of the substrate and zeolite coatings based on previous investigations of MFI coating on titanium alloy. Silicalite-1 is a zeolite composed entirely of pure siliceous material with no potentially hazardous ions such as aluminum in its structure. *In situ*, they created a silicalite-1 coating on the surface of Si (100) wafers. Despite being hydrophobic, the silicalite-1 film has the ability to increase the quantity and rate of proliferation of initially attached human osteoblast-like MG-63 cells. The silicalite-1 layer enhanced cell viability when compared to the standard Si (100) substrate (Doubkova et al., 2019).

### 2.2 Clinoptilolite

Clinoptilolite (CLN) is a widely used natural zeolite with good biocompatibility. It has a three-dimensional crystal porous structure with a general formula of (Na, K)_6_Al_6_Si_30_O_72_•20H_2_O, where the Si/Al ratio can range from 4.0 to 5.3 (Helmi et al., 2022). It is commonly utilized for organism detoxification, medicine delivery, and biosensor creation. CLN also contains anti-oxidant, anti-tumor, anti-inflammatory, and anti-apoptotic properties. Adding metal oxides like zirconia or alumina could strengthen HAP and improve its mechanical performance completely. Al_2_O_3_ is a material that has a strong mechanism, abrasion resistance, and anti-corrosion properties. According to reports, pure bovine hydroxyapatite (BHAP) and coatings mixed with CLN and Al_2_O_3_ are capable of resisting early bacterial adherence and biofilm formation (Popescu-Pelin et al., 2020; Helmi et al., 2022).

In 2016, clinoptilolite/poly (e-caprolactone)-poly (ethyleneglycol)-poly (e-caprolactone) (CLN/PCL-PEG-PCL) composite scaffolds for bone tissue engineering applications were reported. With a consistent solvent-free powder compression/particulate leaching process, scaffolds with variable clinoptilolite ratios (10% and 20%) were created. The presence of CLN may enhance mechanical characteristics. Protein adsorption capacity of scaffolds was also investigated and result showed that CLN/PCL-PEG-PCL scaffolds have a greater adsorption capacity *in vitro*. Furthermore, cell proliferation and osteoblastic differentiation on the scaffolds were studied *in vitro*, and the conclusion was that extremely porous CLN/PCL-PEG-PCL composite scaffolds with effective pore connectivity can operate as osteoinductive carriers for applications in bone tissue engineering (Pazarceviren et al., 2017). Then the *in vivo* effect of the CLN/PCL-PEG-PCL scaffolds for bone tissue engineering was performed in 2019. The scaffolds were assessed in a unicortical bone defect at the anterior of the proximal tibia in adult rabbits after 4 and 8 weeks of implantation for biocompatibility, biodegradation, compressive strength, and induction of osteogenic differentiation *in vitro*. Similar to the findings *in vitro*, scaffolds containing CLN promoted bone tissue regeneration efficiently and in a dose-dependent way. CLN improved high quality bone union, cortical growth, and bone-scaffold interaction at the lesion location. These findings demonstrated that CLN in a composite scaffold promotes bone regeneration and aids in bone repair (Pazarceviren et al., 2017; Pazarceviren et al., 2020). Recently, CLN was utilized as a food supplement to aid with bone development. Angela Fabiano et al. developed a calcium oral controlled-release device based on zeolite for osteoporosis prevention. They created calcium controlled-release particles with CLN and Precirol^®^ that could progressively release calcium in animals. The findings indicated that these particles could improve bioavailability, reduce daily doses, and lessen negative effects. *In vivo* results showed that negative charges on the surface of zeolites might limit calcium ion release, increasing the concentration of zeolites in bone and bone marrow (Fabiano et al., 2019). Zeolites in food could boost the absorption of minerals like calcium and phosphorus, which determine bone strength, if 4% CLN is added to hens with varying characteristics in different groups (Biesek et al., 2021).

Another advantage of CLN is that it does not absorb trace elements such as vitamins or minerals while neutralizing the majority of toxins and eliminating them from the body. A novel composite scaffold with clinoptilolite-nanohydroxyapatite/chitosan-gelatin structure has been developed for being used in bone tissue engineering. Furthermore, the effects of CLN and nano HAP on the physicochemical properties of the scaffold were investigated, including density, mechanical behavior, biodegradation, and biomineralization. In comparison to control scaffold, the addition of CLN and nano HAP results in a greater surface area, higher biomineralization, a reduced rate of degradation, and increased mechanical strength. Importantly, no toxicity in the biological response was tested (Sadeghinia et al., 2020). Pavelic et al. intervened osteoporosis patients for 1 year using a specified cation exchange clinoptilolite named Panaceo Micro Activation (PMA)-zeolite-clinoptilolite. The findings demonstrated that it was safe in humans and could increase bone growth as well as prevent bone resorption following ovariectomy. Furthermore, when compared to the control group, patients treated with PMA-zeolite-clinoptilolite had higher bone mineral density, higher levels of markers suggesting bone growth, significantly lower pain, and significantly higher life quality. Because of the beneficial effects of PMA-zeolite-clinoptilolite on bone integrity and osteoporosis treatment, PMA-zeolite-clinoptilolite has the potential to be a new alternative auxiliary therapy technique for osteoporosis (Kraljevic Pavelic et al., 2021).

### 2.3 Zeolite A

Zeolite A is a sodium aluminum silicate synthesized with the formula Na_12_(AlO_2_)_12_(SiO_2_)_12_·27H_2_O. For a long time, zeolite A has been used to treat bone-related disorders. It has the potential to promote the proliferation, differentiation, and synthesis of TGF-β in normal adult human osteoblast-like cells *in vitro*, as well as to prevent osteoclast-mediated bone resorption. (Keeting et al., 1992; Schutze et al., 1995).

Wang et al. integrated zeolite coatings into 3D printed porous bioactive titanium implants with strontium ions to promote bone growth and osseointegration. When compared to bare titanium alloys, the composite materials demonstrated superior apatite formation, biocompatibility, and corrosion resistance. According to research, zeolite coatings can prevent harmful ions from leaking from titanium in corrosive conditions and improve the biocompatibility of Ti6Al4V implants. The zeolite coatings can further boost osteogenic activity by continuously releasing Sr ions from the composite *via* ion exchange in SBF, as evidenced by ALP activity and mineralization ability. More notably, an *in vivo* test on a rabbit revealed that Sr ions inserted zeolite A coatings on Ti6Al4V may significantly induce new bone formation in and around the pores in the first 4 weeks, resulting in early implant stability. It can be concluded that Sr ions-exchange zeolite A coatings can improve the biocompatibility and osteo-inductivity of porous Ti6Al4V alloys significantly. (Wang et al., 2018).

Zeolite-chitosan composite scaffolds (Chen et al., 2000; Wan Ngah et al., 2012; Yu et al., 2013) and gelatin/zeolite composite scaffolds (Mintova et al., 1999) are known zeolite scaffolds to enhance bone regeneration. To improve oxygen transport inside the scaffolds, freeze-drying zeolite-chitosan composites containing zeolite A were created and found to aid in bone tissue engineering. In comparison to chitosan and other chitosan/zeolite nanocomposites with greater zeolite percentages, chitosan/zeolite with an appropriate zeolite percentage (0.5%) in chitosan provides a suitable substrate for cell adhesion and proliferation. However, a high concentration of zeolite (2%) in chitosan was cytotoxic to the cells. Although zeolite (1%) was comparatively non-toxic, it demonstrated poor cell adherence, viability, and proliferation. Only chitosan/zeolite A (0.5%) nanocomposites achieved a balance between approximately excellent cell adhesion, proliferation, and low cytotoxicity in their investigation. As a result, the optimal zeolite concentration in composites must still be investigated *in vivo* (Akmammedov et al., 2017).

Another study used the calcium forms of zeolite A as a carrier for the bisphosphonate because it has a strong affinity for calcium hydroxyapatite and the capacity to promote bone growth. Furthermore, these calcium ions are exchanged for sodium and potassium ions in the ion exchange process under the effect of bodily fluid. Following this step, the bisphosphonate will lose its contact with the zeolite and be wisely released. According to the findings, A type zeolites have greater ion exchange ability than other zeolites and can accomplish long-term release (Sandomierski et al., 2020).

**FIGURE 6:**
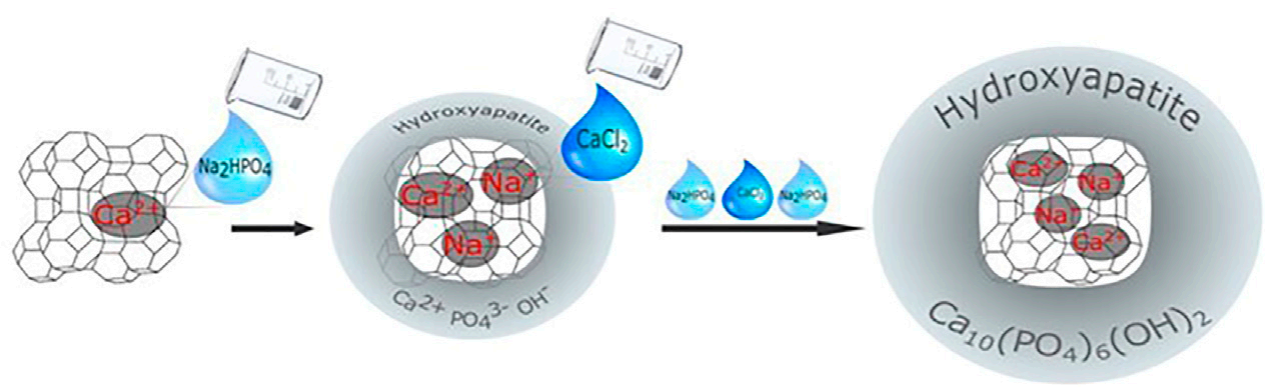
A scheme of the synthesis, ion exchange, and bio-mineralization of hydroxyapatite (Okulus et al., 2019).

Apart from the benefits listed above, zeolite A can also be used as an active filler in dental composites, where it has the potential to promote HAP formation and thus facilitate remineralization. Calcium ions from the environment can also aid in the formation of HAP in the human mouth (Figure 6). The ability of composites containing calcium zeolites and zeolites with the HAP layer to release calcium ions during saline incubation has been demonstrated. The amount of calcium ions released is comparable to or even greater than that of calcium phosphate-filled composite. It could be attributed to the fact that when calcium zeolites are combined with HA, the composition has a higher re-mineralization potential (the ability to release calcium ions) than HAP-containing red blood cells due to the presence of Ca^2+^ -releasing agents (Okulus et al., 2019).

### 2.4 Zeolite Y

Another type of zeolite that can be used as a scaffold is zeolite Y, whose spatial structure contains 12-member ring pores formed by the connection of silicate and aluminate units, and these pores join together to form super cavities (Dehghani et al., 2019). It is likewise beneficial to cell adhesion and proliferation (Tavolaro et al., 2016). The anoxic environment of tissue engineering scaffolds is not conducive to bone formation and tissue repair. Seifu et al. successfully fluorinated zeolite particles as an oxygen transporter for tissue engineering in their investigation. In the presence of fluorinated zeolite particles, the concentration of dissolved oxygen increased significantly. When fluorinated zeolite particles are present, the concentration of dissolved oxygen increases significantly, providing oxygen for scaffolds. The fluorinated particles were evenly embedded in 3D polyurethane scaffolds during the fabrication process, and the presence of the fluorinated zeolite (FZ) particles had no effect on the viability of vascular smooth muscle cells. Furthermore, cell proliferation on the FZ-containing polyurethane (PCU-FZ) scaffolds was significantly higher than on the control PCU scaffolds, and cell depths of infiltration into the PCU-FZ scaffolds were twice as deep as in the PCU scaffolds. PCU-FZ scaffolds, when combined, have the potential to improve oxygen delivery to cells in tissue engineering (Seifu et al., 2011).

HAP has also been widely used as a bone repair material, it is found primarily in teeth and bone and provides rigidity and strength to the bone. However, it has some drawbacks, such as a slower dissolution rate than bone, which may cause patients’ recovery time to be delayed. Furthermore, changing the properties of the traditional form of HAP by adjusting the structure and synthetic method is difficult. Nida Iqbal et al. used a cost-effective microwave-assisted wet precipitation method to successfully synthesize a bioactive composite with HAP and zeolite Y. Biological studies on silica-based materials have shown that these composites can improve the quality and rate of bone tissue repair when compared to HAP alone. They used a cytotoxicity assay to confirm the viability of human osteoblasts on the composites. Furthermore, FESEM results demonstrated that the material allowed cells to adhere to its surface (Iqbal et al., 2014).

### 2.5 Zeolites of other types

Aside from the most regularly utilized zeolites, there are some other zeolites that may be useful in bone tissue engineering. Biomineralization and apatite crystal formation occurred on PLGA/zeolite scaffolds, and mechanical characteristics were improved. These PLGA/zeolite nanoscaffolds have adequate pore size and interconnected canals, superior mechanical characteristics, apatite production, cell compatibility, and good biodegradability due to the porous structure and features of zeolite (Davarpanah Jazi et al., 2017). Porous magnesium-xzeolite (x = 0, 3, 5, and 7 wt%) scaffolds with varying zeolite concentrations (99% purity, particle size <45 μm) were manufactured in a study using a combination of powder metallurgy and space holder processes. According to the findings, introducing zeolite into Mg composite scaffolds increased compressive strength and corrosion resistance over Mg scaffolds without zeolite (Saheban et al., 2018). In the second growth process, zeolite membranes were synthesized on calcium silicate support using W zeolite in two distinct Si/Al ratios as zeolite seed. W zeolite has the ability to promote bone regeneration and can be used in drug delivery (Medina-Ramirez et al., 2018). A group of researchers investigated the properties of zeolite in Zeolite/Collagen (ZC) nanocomposite. They created the bone deformities on the rabbit femur and randomly assigned each animal to one of three groups. A defect was produced in a normal control group (NC) without any intervention, and the skin incision was sutured. They produced the flaw and inserted the ZC nanocomposite into it in the ZC group. They put the HAP into the generated flaw in the HAP group. On day 15, the values of the index of spongiosa were highest in the ZC group and lowest in the HAP and NC groups. It implies that ZC nanocomposite may be suitable for bone repair. The capacity of the zeolite/collagen composites to promote the formation of HAP was also validated in research (Faraji et al., 2019). Researchers examined the influence of zeolite on cell viability, proliferation, osteogenic/odontogenic differentiation, and mineralization in human dental pulp stem cells (hDPSCs) cultivated on PCL-PEG-PCL nanofibers. The findings indicated that PCL-PEG-PCL/Zeolite nanofibrous scaffolds might improve hDPSC cell adhesion, proliferation, and osteogenic differentiation (Alipour et al., 2019). When compared to PCL-PLA nanofiber scaffolds alone with nHAP, nanofiber scaffolds containing PCL, PLA, nHAP, and zeolite were found to have the capability of encouraging the proliferation of hDPSCs for a superior viability and quality of growth (Mohandesnezhad et al., 2020).

Zeolites, in addition to being employed directly as scaffolds or coatings in defective areas, can also be used as a food supplement to aid in bone production. Jameela Banu et al. conducted another study on female C57BL/6 mice, feeding them diets containing zeolite (zeo S and zeo O) or coral calcium for 6 months and examining the bone biochemical markers and cell factors in the blood. The trabecular number and ratio of bone volume to total volume increased after feeding with coral calcium and zeolite, but trabecular separation reduced in other groups. In general, zeolite and coral calcium may help to prevent bone loss after menopause (Banu et al., 2012).

### 2.6 Zeolitic materials

**FIGURE 7:**
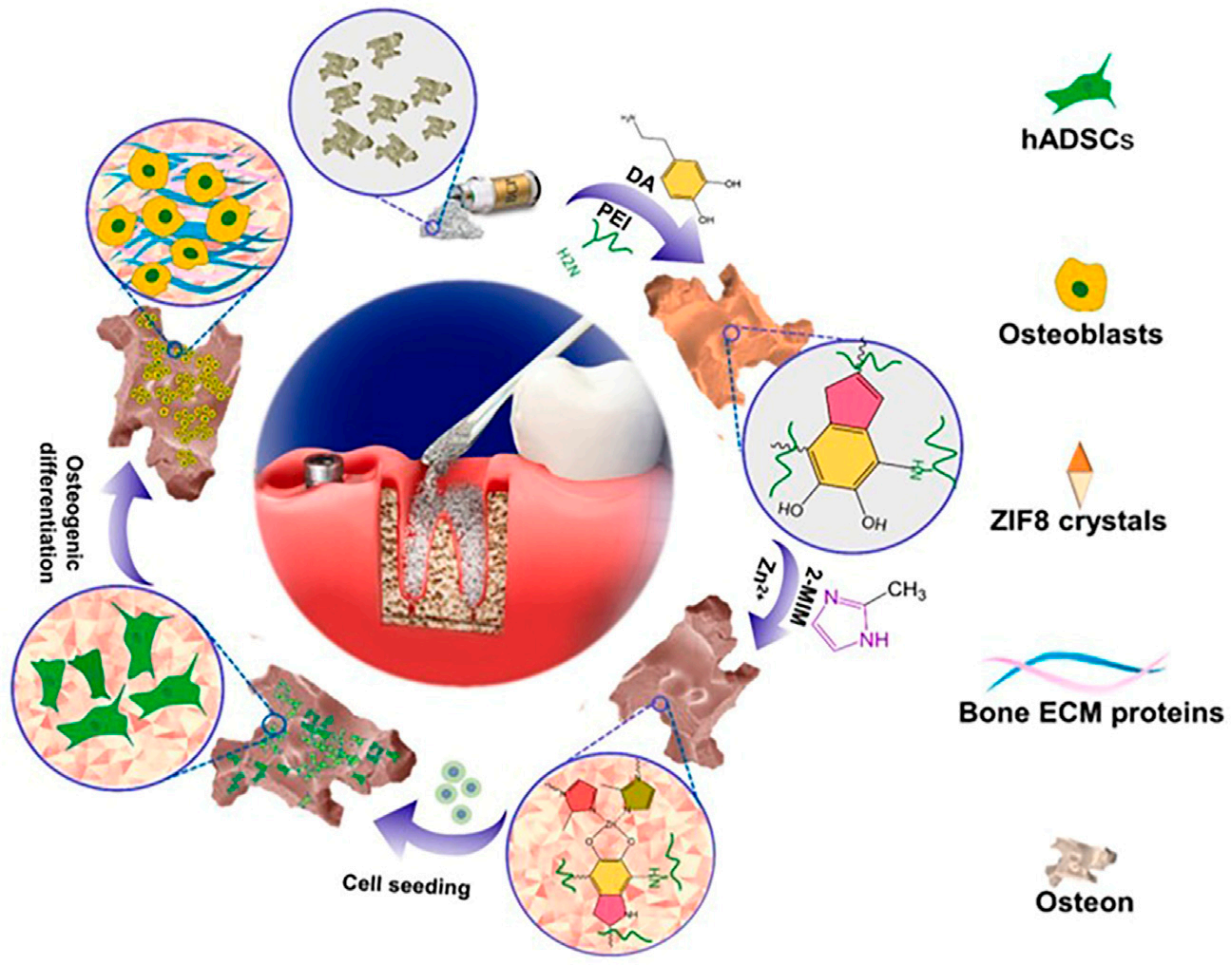
A scheme for the fabrication of ZIF-8-osteon and the process of promoting osteo-conductivity and osteo-inductivity in osteocytes ((Fardjahromi et al., 2021).

Metal-organic frameworks (MOFs) are porous crystalline materials composed of metal ions and organic ligands that have optimal properties such as large surface area, tunable porosity, designable functionality, and high thermal stability (Lian et al., 2017). With the size of 0.34nm, Zn (2-methylimidazolate)_2_ (ZIF-8) is one of the most investigated MOFs. It has a sod topology comprised of 1.16 nm cages connected through six-membered windows. The advantages in microporosity, large surface area, rich structural variety, and high chemical and thermal stability make it broadly used (Lin et al., 2019; Chen et al., 2020; Ozyilmaz et al., 2022). The majority of MOFs either contain cytotoxic elements such as chromium (Karimi-Maleh et al., 2021) or are synthesized using non-acquire based methods, whereas ZIF-8 thin film can be synthesized using acquire based methods (Mohammad et al., 2020; Yang et al., 2020; Fardjahromi et al., 2021; Nankali et al., 2021). Furthermore, because of its benign production conditions and high biocompatibility, ZIF-8 is widely regarded as one of the most preferred MOFs for biomedical applications (Ricco et al., 2018; Feng et al., 2019). ZIF-8 is a MOF subclass that has low cytotoxicity, suppresses bacterial activity, and stimulates osteogenesis (Zhong et al., 2020). ZIF-8’s distinct properties enable it to be employed as a biomaterial in bone tissue engineering. It can be used as a coating or scaffold to aid with bone tissue regeneration. Nano ZIF-8 titanium implants have been used to demonstrate onco-integration. Nano ZIF-8 developed rat bone mesenchymal stem cells into osteoblasts better than ionic Zn at the same Zn^2+^ dose *in vitro* and *in vivo* (Ejeian et al., 2020; Tao et al., 2020; Gao et al., 2021). Its exceptional ability to improve scaffold osteo-conductivity and osteo-inductivity is due to two major factors: The physical and chemical properties of the surface can be altered to promote cell adhesion, proliferation, and differentiation. Second, it has been shown that Zn elements produced during scaffold breakdown are essential for osteoblast growth and can promote bone regeneration (Yang et al., 2020). Besides that, ZIF-8 can also be used to load and release osteogenic differentiation-related bioactive molecules (Tao et al., 2020; Toprak et al., 2021). ZIF-8 can also be utilized to increase mechanical qualities in synthetic polymers as a reinforcing ingredient (Ahmed et al., 2019). Researchers presented a method for enhancing bone formation. They evaluated ZIF-8-Osteon’s capacity to support cell attachment, proliferation, and osteogenic differentiation *in vitro* and *in vivo*, and discovered that ZIF-8 thin-film coating may improve BCP osteo-inductivity and osteo-conductivity. Cell spreading, adhesion, and proliferation can all be improved by the ZIF-8 thin-film coating. After 4 weeks of bone regeneration, *in vivo* testing revealed significant new bone development. The ZIF-8 thin-film coating facilitates modification without the use of toxic reagents or organic solvents (Fardjahromi et al., 2021). Figure 7 depicts the manufacture of ZIF8-Osteon as well as the enhancement of the Osteon’s osteo-inductivity and osteo-conductivity (Fardjahromi et al., 2021).

Another study found that ZIF-8 thin-film coating can efficiently stimulate cell adhesion and proliferation in both dynamic and static cell culture conditions by improving PS bead surface attributes such as roughness, wettability, surface free energy, and surface charge. *In vitro*, ZIF-8 thin-film coating for bone implants can influence microcarrier surface characteristics and improve stem cell adhesion and proliferation. The level of autocrine cell fate determination markers in the ZIF-8 modified polypropylene membrane was also raised. When compared to the tissue culture plate, osteogenic markers were clearly overexpressed in response to ZIF-8 layer biomechanical features (Asadniaye Fardjahromi et al., 2020; Ejeian et al., 2020). The nanoscale ZIF-8 coating on porous titanium implants increases osteogenic-related gene expression and protein secretion, as well as extracellular matrix mineralization, osteoblastic activity, and bone regeneration (Chen et al., 2017). By releasing Zn^2+^, the ZIF-8 also had antibacterial effects against *S. aureus* and *E. coli*. *In vivo*, the ZIF-8 implant also demonstrated strong osseointegration and antimicrobial activity. Overall, ZIF-8 provides an alternative method for producing multifunctional Ti implants that can aid in the management of implant-associated infections (Tao et al., 2020; Toprak et al., 2021).

**FIGURE 8:**
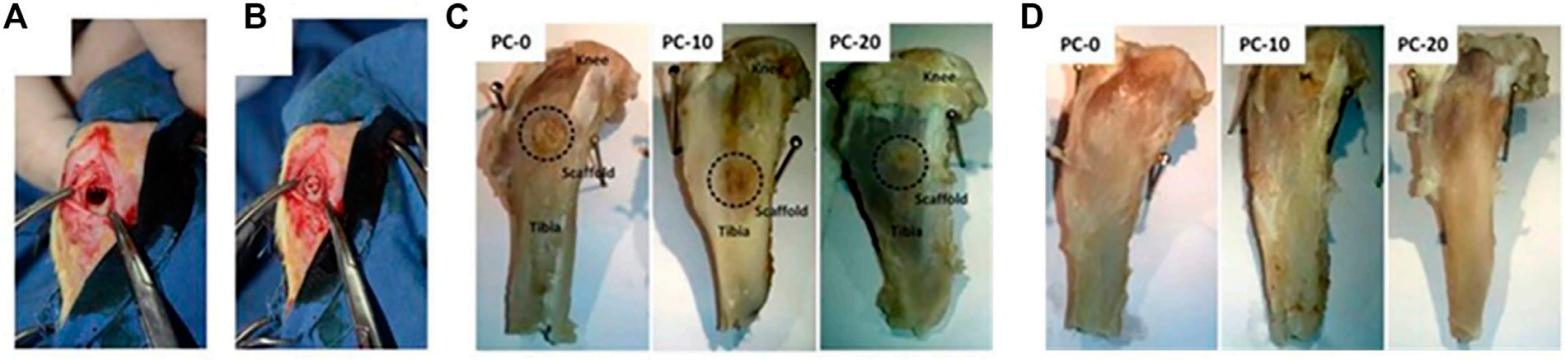
**(A)** Defected uni-cortical tibial, **(B)** after implantation of a scaffold in PC-20 group, **(C)** tibia at the end of fourth week, and **(D)** tibia at the end of eighth week after implantation (Pazarceviren et al., 2020).

The nanoscale zeolitic imidazolate framework-8 (nanoZIF-8) zeolite composite scaffold demonstrated increased mechanical strength, a similar structure with strongly connected macropores, and the ability to sustainably preserve physic properties capable of bearing the strength of implant sites. Linna Zhong et al. employed 3D printing extrusion to introduce nanoZIF-8 into polycaprolactone (PCL) and dicalcium phosphate dihydrate (DCPD) composite scaffolds to increase mechanical strength. They may also stimulate osteogenic differentiation of stem cells *in vitro* without the use of any other osteogenic agent and can steadily improve elastic modulus (Hao et al., 2019; Pazarceviren et al., 2020). As shown in Figure 8, uni-cortical tibial defects treated by scaffolds with zeolite in different concentrations have been compared. PCL, DCPD, and nanoZIF-8 were used to create the porous composite scaffold PCL/DCPD/nanoZIF-8. *In vitro*, BMSC osteogenic differentiation into osteoblasts was enhanced by the PCL/DCPD/nanoZIF-8 porous composite scaffold, and their response to osteogenic differentiation-associated genes and proteins was detected. 3D printed PCL/DCPD/nanoZIF-8 porous scaffolds mended rabbit calvarial lesions faster than non-nanoZIF-8 porous scaffolds, according to *in vivo* experiments (Zhong et al., 2020).

Similarly, RIS@ZIF-8 nanoparticles have showed potential in terms of increasing the expression of osteogenic genes (ALP, OPG, and Runx2) as well as anti-resorption properties, which could be employed to treat bone defects locally (Cheng et al., 2020). The ability of zeolite scaffolds to stimulate osteogenesis may be related to the presence of soluble silica in zeolite scaffolds, which promotes osteogenesis by boosting osteogenesis and decreasing osteoclastogenesis while encouraging collagen type 1 formation and osteoblast differentiation (Kraljevic Pavelic et al., 2021). In addition, zeolite contains silicon, which has previously been related to bone synthesis in studies. Si and Al have the potential to promote caryocinesia in the bones (Saheban et al., 2018). Zeolite scaffolds, due to their high biocompatibility, can assure appropriate blood and nutrient supply to the bone defect area, hence encouraging rBMSC osteogenic development. The study discovered that the zeolite/collagen group had the highest sponge index when compared to the control groups. As a result, they can aid in the reconstruction of bone deformities (Faraji et al., 2019). CA-CS/Z multifunctional hydrogels were developed to maintain the bone transplant environment, ensure blood supply, improve osteogenic differentiation, and accelerate bone regeneration *in vivo*. CA-CS/Z also improved vascularized osteogenesis by stabilizing bone graft materials and hastening bone regeneration and wound healing (Liu et al., 2020).

## 3 Conclusion and future perspective

This review was written to provide researchers with a concise summary of the properties and uses of zeolites in bone tissue engineering. Both naturally occurring and artificially produced zeolites are porous minerals with properties that make them well suited to and beneficial in the field of bone tissue engineering. It is possible to tailor zeolite’s pore shape, pore size, and element content to produce a wide range of scaffolds. The outstanding biocompatibility and acceptable physical and chemical properties of zeolites make them useful in a wide variety of biomedical applications, including drug delivery, scaffolds for cell adhesion. Furthermore, as a coating for metal implants and a bone tissue scaffold for repairing large-scaled bone tissues, zeolite has the ability to insist corrosion and enhance the elastic modeling. Besides that, dietary supplement application for zeolite was also present great application potential.

Although zeolite has been used extensively in recent studies, further details must be resolved before it can be used in therapeutic settings. In the first place, there is the structure-function relationship, which can be used as a deciding element when comparing the bio-effects of different types of zeolites. Studying the effects of varying Si/Al ratios and crystal structures on bone regeneration performance is crucial. Researchers also establish the molecular mechanism *via* which zeolite induces osteoblast differentiation. This will be used as a blueprint for future zeolite creation. Second, the zeolite’s aluminum content raises questions about the material’s long-term biosafety; further research is necessary with regards to the effects of Al on bone and other vital organs. Third, zeolite supplements offer a novel route for zeolite’s administration, whereas broader systemic benefits remain elusive, such as the impact on gut microbes and the metabolic mode of these organisms. These issues provide us with fresh avenues for investigation into the biomedical uses of zeolites.
